# Variability in thermal and phototactic preferences in *Drosophila* may reflect an adaptive bet‐hedging strategy

**DOI:** 10.1111/evo.12813

**Published:** 2015-11-30

**Authors:** Jamey S. Kain, Sarah Zhang, Jamilla Akhund‐Zade, Aravinthan D. T. Samuel, Mason Klein, Benjamin L. de Bivort

**Affiliations:** ^1^ Rowland Institute at HarvardCambridgeMassachusetts02142; ^2^ Center for Brain ScienceHarvard UniversityCambridgeMassachusetts02138; ^3^ Department of Organismic and Evolutionary BiologyHarvard UniversityCambridgeMassachusetts02138; ^4^ Department of PhysicsHarvard UniversityCambridgeMassachusetts02138; ^5^ Department of PhysicsUniversity of MiamiCoral GablesFlorida33124

**Keywords:** Evolutionary strategy, heritability, personality, phototaxis, thermotaxis, variation

## Abstract

Organisms use various strategies to cope with fluctuating environmental conditions. In diversified bet‐hedging, a single genotype exhibits phenotypic heterogeneity with the expectation that some individuals will survive transient selective pressures. To date, empirical evidence for bet‐hedging is scarce. Here, we observe that individual *Drosophila melanogaster* flies exhibit striking variation in light‐ and temperature‐preference behaviors. With a modeling approach that combines real world weather and climate data to simulate temperature preference‐dependent survival and reproduction, we find that a bet‐hedging strategy may underlie the observed interindividual behavioral diversity. Specifically, bet‐hedging outcompetes strategies in which individual thermal preferences are heritable. Animals employing bet‐hedging refrain from adapting to the coolness of spring with increased warm‐seeking that inevitably becomes counterproductive in the hot summer. This strategy is particularly valuable when mean seasonal temperatures are typical, or when there is considerable fluctuation in temperature within the season. The model predicts, and we experimentally verify, that the behaviors of individual flies are not heritable. Finally, we model the effects of historical weather data, climate change, and geographic seasonal variation on the optimal strategies underlying behavioral variation between individuals, characterizing the regimes in which bet‐hedging is advantageous.

How do organisms thrive in the face of fluctuating environmental conditions? Understanding their strategies is a major challenge in evolutionary ecology. One versatile adaptive “solution” is phenotypic plasticity—in which an individual adjusts its phenotype in direct response to the current environmental condition, such as modulation of leaf size in response to lighting conditions (Sultan 2000). In principle, plasticity can embody perfect solutions to any environmental challenge, as animals can employ a “lookup table,” producing the perfect response to any condition. However, there are limitations to plasticity (DeWitt et al. 1998; Murren et al. 2015), such as the metabolic cost of encoding a lookup table, and the speed with which an organism can change its phenotype. The latter constraint, phenotypic inflexibility, applies particularly to animals, such as insects, that attain a final adult life stage. That said, behavioral phenotypes specifically have the potential to be quite flexible.

Populations can also survive changing conditions by having diversified phenotypes as a result of genetic variation; this also allows organisms to readily evolve/adapt to new conditions. This is termed “adaptive tracking.” However, if the environmental changes are transient, as one would observe with seasonal variation, it would be detrimental to rapidly adapt to their local/temporal environment (summer adapted animals would not fare well during the winter). Instead, an adaptive response to fluctuating selection can be to suppress the phenotypic expression of genetic variation, reducing heritability (Kawecki 2000). Although genetic variation can be maintained under some circumstances, recent evidence suggests that temporal environmental fluctuations may reduce polymorphism through most of the genome more severely than even constant environments (Huang et al. 2014).

A third possible solution to the problem of uncertainty is to use a bet‐hedging strategy (also called risk‐spreading), in which developmental stochasticity produces a distribution of adult phenotypes. In diversified bet‐hedging, a single genotype can (stochastically) generate a distribution of phenotypes, guaranteeing that at least some individuals are well suited to any environmental condition (Hopper 1999; Simons 2011; Levy et al. 2012). More formally, bet‐hedging can be defined as evolutionary strategies that reduce the variance in fitness (maximizing the geometric mean of fitness, at the expense of the arithmetic mean of fitness) across time and environmental conditions.

Some individuals in bet‐hedging populations will have reduced fitness for any given environmental condition. The adaptive value of bet‐hedging increases with increased environmental variation (Haccou & Iwasa 2002), provided that the fluctuations are not brief compared to animal life spans (Müller et al. 2013). An elegant example is the timing of seed germination (Cohen 1966). If all the seeds from a desert plant germinated after the first rain of the season, they would be vulnerable to extinction if there is an extensive drought before the second rain. Conversely, if the seeds all germinate later in the season, they will be at a disadvantage relative to other seeds that had germinated at the first opportunity (in typical seasons without an early drought). Thus, an optimal strategy may be for the plant to hedge its bets and have a fraction of seeds delay germination while the others respond to the first rain. Of course, this is biology, and real organisms surely employ a combination of plasticity, adaptive tracking, and bet‐hedging (Svardal et al. 2011). Yet, bet‐hedging in animal systems remains poorly studied, in part because of the difficulties of studying intragenotypic variability within a common environment, let alone in more complex and biologically realistic scenarios.

The evolutionary optimality of bet‐hedging can explain why a single genotype gives rise to a distribution of phenotypes (Sasaki & Ellner 1995). This question has also been addressed within behavioral ecology from the perspective of animal personality. Genetic variants are often assumed to underlie the behavioral differences described as personality variants, and indeed animal personality syndromes may be largely heritable (up to 52% of variance; Dochtermann et al. 2015). However, to explain the remaining variance in individual behavior, stochastic mechanisms generating intragenotypic variability are almost certainly at play, including bet‐hedging. Thus, in explaining variation in the personality of individual animals, it is essential to assess the degree to which bet‐hedging is itself under genetic control.

Although animal personality is typically evaluated along axes that correspond to dimensions of variation in human personality, such as shyness versus boldness, behavioral variation is richly multidimensional (Ayroles et al. 2015). We assert that if there is (1) variation in a behavior among closely related individuals, and (2) these idiosyncratic differences persist within the lifetime of those individuals, this is an example of a facet of animal personality, broadly construed. As an example, fruit flies exhibit lifelong locomotor biases (preferring to turn left or right on an individual‐by‐individual basis; Ayroles et al. 2015; Buchanan et al. 2015). This variation has no clear relationship with the bold‐shy axis, but represents one orthogonal axis of “personality” among many.

Fruit flies are one of the most studied organisms for many aspects of biology, including the basis of behavioral diversity. We chose to study bet‐hedging using the fly's positional response to thermal gradients (thermotaxis) and spatially varying illumination (phototaxis). The thermotactic and phototactic responses of *Drosophila* depend on a wide range of environmental and stimulus parameters (Dillon et al. 2009), such as humidity (Waddington et al. 1954), directionality of the light source (Rockwell & Seiger 1973), and agitation state of the flies (Lewontin 1959; Rockwell & Seiger 1973; Seiger et al. 1983). The type of phototactic response is particularly sensitive to the state of agitation. In most *Drosophila* species, agitated animals exhibit “fast phototaxis” toward the light source, whereas unagitated animals exhibit “slow phototaxis” as a preference to stay in shaded areas. The former response is thought to reflect a predator evasion instinct to move skyward (Scott 1943), whereas the latter reflects a thermoregulatory and antidesiccation instinct during rest (Pittendrigh 1958).

Thermal experience has dramatic effects on the life history of *Drosophila* (Miquel et al. 1976; Ashburner 1978.; Ashburner et al. 2005). Individuals can control this experience through a variety of behaviors (Parry 1951; Digby 1955) including shade‐seeking phototaxis and direct positional response to thermal gradients. Thus, the net resting behavior of flies will greatly affect the amount of heat they experience across their lifetime, and consequently their vulnerability to unusual weather, season, and climate fluctuations. The light versus shade and thermal gradient resting preferences of animals can be readily quantified in laboratory experiments.

Recent results from several groups hint that fluctuating temperature specifically could favor bet‐hedging. The optimal preferred temperature of ectotherms may not be the single temperature that yields the fastest growth, if the fitness function on temperature is skewed (Martin & Huey 2008). Selection for heat resistance indirectly increased cold resistance (Condon et al. 2015), suggesting that evolutionary solutions to extreme temperatures may act on the absolute deviation from mean temperatures as much as the direction of deviation. Moreover, populations evolved specifically in fluctuating environments acquired thermal resistance to temperatures outside the selected temperature range, even when the fluctuating temperatures were moderate, and centered on the animals’ preferred temperature (Condon et al. 2015; Tobler et al. 2015).

We found considerable variation in the slow phototactic and thermotactic responses of very recently domesticated *D. melanogaster* from Cambridge, Massachusetts. Some individual flies strongly preferred to rest in the shaded portion of the phototactic arena (or the cool portion of the thermotactic arena), others strongly preferred the lit portion (or the warm portion). We wondered whether this behavioral diversity represented a bet‐hedging strategy to maximize fitness in the face of fluctuating seasonal or weather conditions. To compare the performance of bet‐hedging versus a strategy in which the individual behavioral preferences are heritable (i.e., adaptive tracking sensu Simons 2011), we developed a model incorporating our behavioral data with local weather and climate data from historical records. Phenotypic plasticity in response to environmental fluctuations is unlikely to explain the behavioral differences we observed between individuals reared in essentially identical laboratory environments; under phenotypic plasticity, we would expect animals to adopt similar behaviors as their response to a similar environment, but this is not what we observe. Our scope here is to specifically consider a head‐to‐head comparison of bet‐hedging and adaptive tracking strategies, both of which remain plausible explanations of the observed behavioral variation. Thus, we test the hypothesis that the observed individual behavioral differences reflect a bet‐hedging strategy, rather than genetic variation underlying an adaptive‐tracking strategy.

We find that the bet‐hedging strategy generally outcompetes adaptive tracking. Because the generation time of *Drosophila* is short relative to the seasons, seasonal temperature fluctuations can induce genetic adaptations in the spring (Bergland et al. 2014), which could then decrease fitness in the summer. This reversal of selective pressures throughout the year renders adaptive tracking counterproductive. The alternative bet‐hedging strategy is particularly valuable when there is high fluctuation in temperature throughout the season. Adaptive tracking is advantageous, however, during seasons that are consistently warm or cold throughout, because it engenders long‐term changes to average behaviors by altering genotypic frequencies. Interestingly, because global climate change will bring about an increase in mean temperatures, we predict that the optimal strategy will change in approximately 100 years, and adaptive tracking will become more advantageous than bet‐hedging.

## Methods

### BEHAVIOR

The *Drosophila melanogaster* line CamA was established from a single, mated female caught from the wild in Cambridge, Massachusetts and propagated in the laboratory for approximately two generations at typical *Drosophila* culture densities prior to behavioral testing. The line inbred CamA was derived by 10 generations of sibling‐pair matings. All flies were cultured on standard growth medium (Scientiis) in 25°C incubators at 30–40% relative humidity on a 12‐h light:12‐h dark cycle. Phototactic experiments were conducted at 23°C. Both behavioral assays were conducted at 30–40% humidity in environmental rooms. We found no difference in the behavioral responses of males versus females and merged their data. For both assays, only those flies registering 10 or more choices were analyzed. (Flies with only a small number of choices yield noisy estimates of individual preference.)

**Figure 1:**
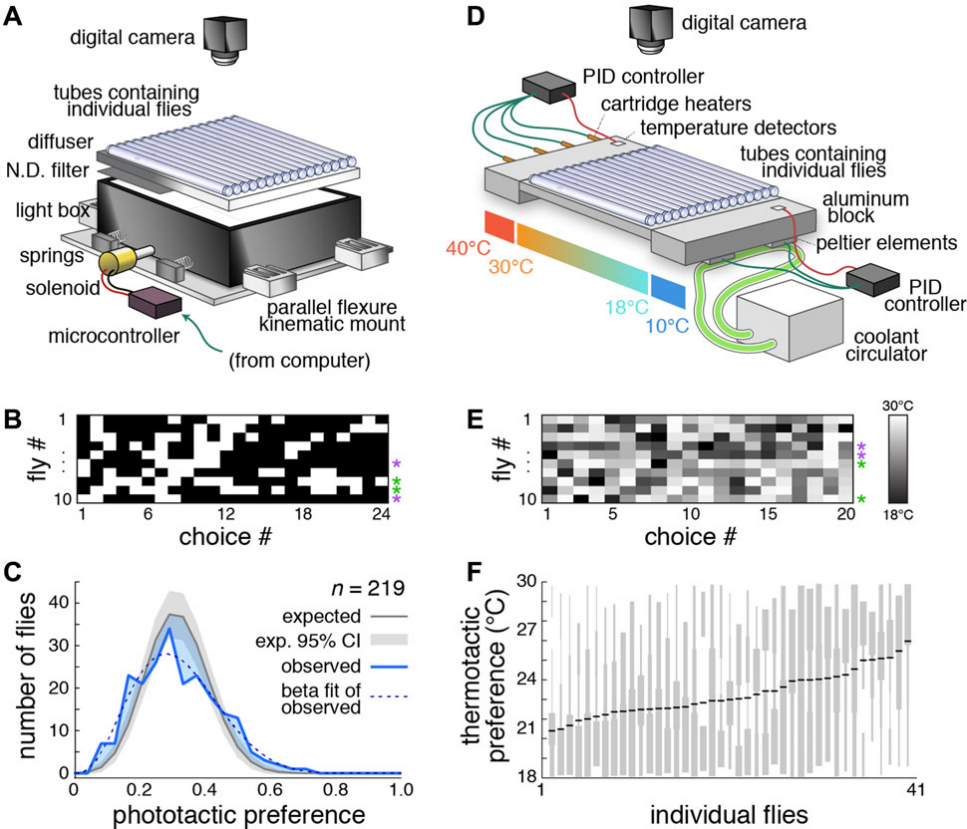
Measurement of phototactic and thermotactic variation and a model of their effect on fitness. (A) Schematic of the “slow photobox”—a device for the high‐throughput characterization of slow phototaxis. Animals were placed individually into clear tubes with a lit and shady side. Their position in the tube was recorded by a camera. (B) Example of data from the slow photobox. Each row represents an individual fly's phototactic preferences at 24 instances, spaced at 10 min intervals. White boxes indicate lit choice and black boxes indicate shaded choice. Purple and green asterisks indicate examples of shade‐ and light‐preferring individuals, respectively. (C) Observed histogram of the phototactic preference across individual flies (blue line). Dashed gray line indicates a best‐fit beta‐binomial distribution for the observed data. Gray line indicates expected distribution for the same flies if they were each to choose light with identical probabilities. Gray shaded region indicates 95% confidence interval of the expected distribution given sampling error. Shaded blue areas indicate discrepancies between the observed and expected histograms consistent with behavioral heterogeneity. (D) Schematic of the “slow thermobox.” (E) Example data from the slow thermobox, as in (B). Grayscale indicates thermotactic preference over time. Purple and green asterisks indicate examples of cool‐ and warm‐preferring individuals, respectively. (F) Histograms of thermotactic preference values across all trials (vertical, gray) for individual flies, sorted by mean preference (black bars).

Age‐ and sex‐controlled flies were placed singly into 30 tubes in the “slow photobox,” which is illuminated from below by diffused white LEDs (5500K, LuminousFilm; Fig. 1A). A 50% neutral density filter was used to generate a lit half and shaded half for each tube. The lit portion of the arenas were slightly (0.1–0.5°C) warmer than the shaded portion. The arenas, illuminator, and diffusers are mounted on kinematic flexure mounts allowing approximately 1 cm translation perpendicular to the testing tubes, under the control of a solenoid/microcontroller system driving vibration at 20 Hz. Agitation of the animals induced them to run and thereby reset their position between successive measurements of their light/shade preference. Each trial consisted of agitation (three 2 sec pulses, each separated by a 1 sec pause), an interval of 577 sec, acquisition of the photo used to score animal position, and a 15 sec interval completing the 10‐min trial. Animal position was determined by subtracting the background image of the rig and calculating the centroid of all pixels that had changed relative to the background (on a tube‐by‐tube basis), subject to a noise‐eliminating threshold.

The slow thermobox (Fig. 1D) was fabricated by placing the acrylic tray of choice tubes used in the slow photobox down on a slab of aluminum with thermal grease. The aluminum slab was in contact with two larger aluminum blocks, one warmed to 40°C with resistive heating elements, and one cooled to 10°C with thermoelectric coolers (Peltier elements). The temperature of both larger blocks was held constant by proportional‐integral‐derivative controllers reading insulated resistance temperature detectors (three‐wire, 100 ohm). The 30–18°C gradient achieved within the choice tubes was measured using an infrared thermometer gun and was highly linear. For each of 20 trials, animals were first agitated by flowing air into the choice tubes, dislodging the animals toward the warm end. After 9.5 min, the tubes were photographed and the position of each animal measured digitally.

Day‐to‐day persistence of phototactic preferences was measured in a modified apparatus in which the floors of the imaging tubes were open at either end onto a surface of standard fly food poured in an approximately 0.5 cm thick layer. This way, the flies could feed during an extended 40 h trial. Day‐to‐day persistence of thermotactic preference was measured by the standard assay, individual housing of flies overnight, and retesting under the standard protocol.

### TEMPERATURE MEASUREMENT

Temperature differences between sun and shade were measured using an infrared thermometer gun on partly cloudy days in the summer and autumn. In one set of comparisons, we measured the temperature of substrates in the shade of clouds, and then waited until approximately 5 min after the cloud had passed and measured their temperature in sunlight. In another set of comparisons, we compared adjacent sunlit and shaded (e.g., by a building or road sign) substrates of the same orientation. Measured substrates included grass, brick, pine branches, tree bark, gravel, etc.

### RAW DATA AND CODE

All raw data used in this study, as well as all code used for data acquisition, statistical analysis, and modeling are available at http://lab.debivort.org/variability-may-reflect-bet-hedging/.

### STATISTICS

Data from individual flies that did not move upon agitation for 3 or more successive trials were discarded because these measurements were clearly nonindependent from trial to trial. Sequential slow phototactic choices were found to have an average of 0.054 bits of mutual information across individuals, indicating effective independence (0 bit indicates complete independence in every animal, 1 complete dependence). This justifies treating behavioral choices as independent events, and shows that the agitation protocol succeeded in rousing the animals between trials. We therefore modeled the expected distribution of light choices with a binomial distribution with parameter *p* equal to the average light‐choice probability of all animals tested, and parameter *n* equal to the number of trials, 24.

### MODELING

**Figure 2:**
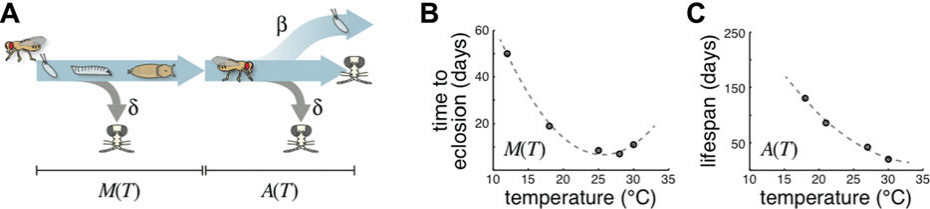
A fly temperature‐dependent life‐history model. (A) Diagram of the fly life‐history model, see description in text. β, birth rate; δ, death rate; *M*, metamorphosis time; *A*, adult life span; *T*, thermal preference index. “Fly skull and crossbones” icons indicate death. (B) Time to eclosion plotted as a function of temperature, as used by the model. Datapoints from Ashburner (1978.). (C) Life span plotted as a function of temperature, as used by the model. Datapoints from Miquel et al. (1976).

See Results and Figure 2 for descriptions of the model. In the bet‐hedging implementations of the model, each fly was randomly assigned a thermal preference index drawn from the experimentally observed preference distribution (fit by a beta distribution; Fig. 1C). In adaptive‐tracking implementations, the seed population was initialized in that way, but all subsequent animals were assigned a preference identical to their mother's preference (thus the model is asexual). Stochastic simulations of finite populations were seeded with 100 flies with ages uniformly distributed on [*M*(*T*), *A*(*T*)]—respectively, the temperature *(T)*‐dependent mean ages of eclosion and death—because flies may overwinter as adults (Izquierdo 1991). We also implemented a version of the model in which the seed population was synchronized to the egg stage. This model was qualitatively indistinguishable. Flies in this initial population were assigned to have developed at random in the sun versus the shade with a probability equal to the population mean thermal preference index. Individual flies were simulated, removed from the virtual population at random according to the parameter δ, and born stochastically at a rate β from mature flies already in the population. The temperature experience of fly *i* on day *j* was determined as *p_i_* × *shadeDiff* × *cloudCover_j_* + *T_j_*, where *p_i_* is the thermal preference index of fly *i*, *shadeDiff* is the temperature difference between light and shade, *cloudCover_j_* is the average fraction of cloud cover on day *j*, and *T_j_* is the in‐shade temperature on day *j*. The birth and death rate parameters were identified (by grid search or hill‐climbing algorithm) as the unique pair of values that satisfy two assumptions: (1) the fly population neither grows nor diminishes across the breeding season, that is, it is at numerical equilibrium, and 2) the mean thermal preference index does not evolve across the breeding season, that is, flies are adapted to typical conditions. For every distinctive weather model, parameter fitting was independently performed using the adaptive tracking implementation. See Table S1 for parameter values.

A related version of the model, which simulates infinite population sizes, was implemented analogously using a system of difference equations, but could be used to efficiently evaluate historical and simulated daily temperature deviations and cloud‐cover values (see Supporting Information for details). In this implementation of the model, clouds reduced the maximum ambient temperature difference attainable by individual flies in proportion to the mean daily cloud cover fraction. Historical daily temperature deviations were normally distributed, and modeled using a 30 parameter autoregression filter of normally distributed white noise. Random cloud cover was generated by drawing a season‐long sequence of values from the observed (non‐Gaussian) distribution of cloud cover fractions. These values were then shuffled until the new cloud cover sequence was no longer correlated with the original sequence (*r* < 0.1), under the constraint that the autocorrelation of the simulated sequence was correlated to that of historical cloud data with *r* > 0.998, thus preserving temporal statistical structure of the sequence. Historical cloud and temperature deviation data were uncorrelated (*r* = 0.02), so simulated sequences of these variables were derived independently.

## Results

### *DROSOPHILA* EXHIBIT MORE BEHAVIORAL VARIABILITY THAN EXPECTED BY CHANCE ALONE

We sought to directly measure the slow phototactic and thermotactic response of recently domesticated *D. melanogaster* flies, and assess to what extent there was individual‐to‐individual variability in this behavior. An isofemale line (CamA) was established from a single fertilized female caught in Cambridge, Massachusetts. To assess phototaxis, sexed and age‐matched CamA adults, cultured on standard fly media, were assayed individually in our “slow photobox” (Fig. 1A), where their light versus shade preference was measured by automated image analysis 24 times per fly (Fig. 1B), once every 10 min. We tested 219 individuals in total, and found that their average light‐choice probability was 0.32 with a SE of 0.032, indicating a preference for resting in the shade. The observed distribution of light‐choice probabilities was considerably overdispersed compared to the null hypothesis that all animals were choosing the light with identical probabilities of 0.32 (*p* = 4 × 10^−6^, 1 × 10^−11^ and < 0.001 by Kolmogorov‐Smirnov [KS] test, χ^2^ test of variance, and bootstrap resampling, respectively; Fig. 1C), indicating considerable individual‐to‐individual behavioral variability. We estimated 44.2% of the experimental variance was due to individual differences, corresponding to a preference index SD across individuals of 0.085 (95% CI = [0.74, 0.94], estimated by bootstrap resampling). These results are similar to our previous findings on agitated phototaxis where we observed significant individual‐to‐individual variability that was not explainable by differences in age, sex, reproductive status, birth order, social interactions, or previous exposure to light (Kain et al. 2012).

To assess thermotaxis, similarly cultured animals were tested individually on a linear thermal gradient (Ryu and Samuel 2002) ranging from 30°C to 18°C (Fig. 1D), which spans most of the range of flies’ natural environment. The position of each of 41 flies within this gradient was measured 20 times per animal, once every 10 min, with their position indicating their per‐trial thermotactic preference (Fig. 1E). The mean average preference was 23.1°C with a SE of 0.22°C. We observed considerable interindividual variation in mean thermotactic preferences (*F* = 3.07, df = 40, *p* < 10^−6^ by one‐way analysis of variance [ANOVA] on fly identities; Fig. 1F). We estimated 14.7% of the experimental variance was due to individual differences, corresponding to an SD across individuals of 1.4°C (95% CI = [1.15, 1.77], estimated by bootstrap resampling).

We performed day‐to‐day persistence experiments to see if the individual differences in thermotaxis and phototaxis were stable across time, rather than arising from transient state differences such as satiety. Individual scores for both phototactic and thermotactic preference were significantly correlated across 24 h inter‐test intervals (Fig. S1; *r* = 0.71, *P* < 0.0001, df = 70 and *r* = 0.48, *P* = 0.002, df = 36, respectively).

### A MODEL TO COMPARE ADAPTIVE TRACKING AND BET‐HEDGING STRATEGIES

Could the observed behavioral individuality represent a bet‐hedging strategy to increase the probability that at least some individuals will be well adapted to the current weather conditions? To test this, we proposed a model of fly development and reproduction (Figs. 2A and S2.) in which an individual animal's behavior could be treated either as perfectly inherited from the mother (i.e., adaptive tracking [AT]), or as nonheritable/stochastic variation indicative of a bet‐hedging strategy (BH). Holding the magnitude of variation constant, we can evaluate which is more advantageous, adaptive tracking, or bet‐hedging, and under what conditions.

In considering how thermal experience might affect fitness, we recognized that the metamorphosis time from egg to adulthood depends on the temperature experienced during that period, in a relationship determined by previous experimental work (Ashburner 1978.; Ashburner et al. 2005), with flies developing fastest at 25°C (Fig. 2B). The expected total life span of flies also depends on temperature (Miquel et al. 1976), with flies living considerably longer at cooler temperatures (Fig. 2C). We assume that the effective temperature experienced throughout adulthood depends on the integrated results of many behavioral choices for each individual fly. By contrast, we assume the temperature experienced during growth from egg through pupa depends on the thermal preference index of each fly's mother (the alternative, that developmental temperature depends on progeny preference, yields qualitatively identical results). These are clearly simplifying assumptions—the total amount of thermal energy integrated across a life span and the choice of oviposition site depend on more behaviors than just phototaxis and thermotaxis. But, constraining the model with empirical data on these behaviors allows us to investigate their roles in fitness. We lastly assume that throughout metamorphosis and adulthood, flies face a constant risk of death (by, e.g., predation, disease, fly swatter, etc.), and after reaching adulthood, flies produce new offspring at a constant rate. Thus, temperature choices represent a trade‐off for the fly (warm‐preferring animals will have the benefit of faster development at the cost of shorter life span) the kinetics of which are temperature‐dependent.

To formulate a single variable representing the diversity of temperature experience due to all dimensions of behavioral variability, we compared our phototactic and thermotactic observations. The effect of phototactic preference on temperature experience depends on the temperature difference between shade and sunlight. This in turn depends on numerous factors, including weather conditions, latitude, season, wind, substrate composition, and duration of exposure to the sun. We measured this directly and determined that a 7°C difference between sun and shade was attained quickly after exposure to sunlight on both natural and artificial substrates in Cambridge, Massachusetts. This estimate is well within the range of previous estimates of the temperature difference between insects in sunlight versus shade (Parry 1951). We observed that the mean light‐choice probability of flies in the slow phototaxis assay was 0.32, with a SD of 0.13 (Fig. 1C). Assuming that a fly which spends *x*% of the time in the light would spend *x*% of the time in the sun and 7°C warmer than the remaining 100 − *x*% of the time, this phototactic variance implies an SD of temperature experience of 0.89°C. The mean observed thermotactic preference was 23.1°C with an SD of 1.4°C. These two observations are in agreement that individual flies have substantially different temperature experiences. For the model, we let the “thermal preference index” of individual flies, integrated across all behaviors determining temperature experience, vary from 0 to 1, corresponding to a 7°C temperature range. This index has the same range as the phototactic data. Allowing it to follow the same distribution across flies as the phototactic data (beta‐distributed, with mean 0.32 and variance 0.22), it reflects a conservative estimate of thermal experience variability, compared to the direct thermotactic measurements.

The model contains two unknown parameters, the lifelong risk of death (δ) from causes other than thermal experience‐dependent mortality, and the birth rate (β) at which new eggs are laid by sexually mature flies in the wild. We have no empirical data from which to assert these values, but the behavior of the model constrains them under two reasonable assumptions: (1) that the population size of flies is the same at the end of each season as the beginning, and (2) that the mean thermal preference index of the population is the same at the end of the season as the beginning, that is, they are adapted to average conditions. These assumptions constrain the random death probability of flies in the wild to 0.013–0.044/day, and the birth probability to 0.037–0.11/mother/day (depending on which weather model is used; Table S1; see Methods for details); both of these ranges seem plausible.

### BET‐HEDGING OUTPERFORMS ADAPTIVE TRACKING

**Figure 3:**
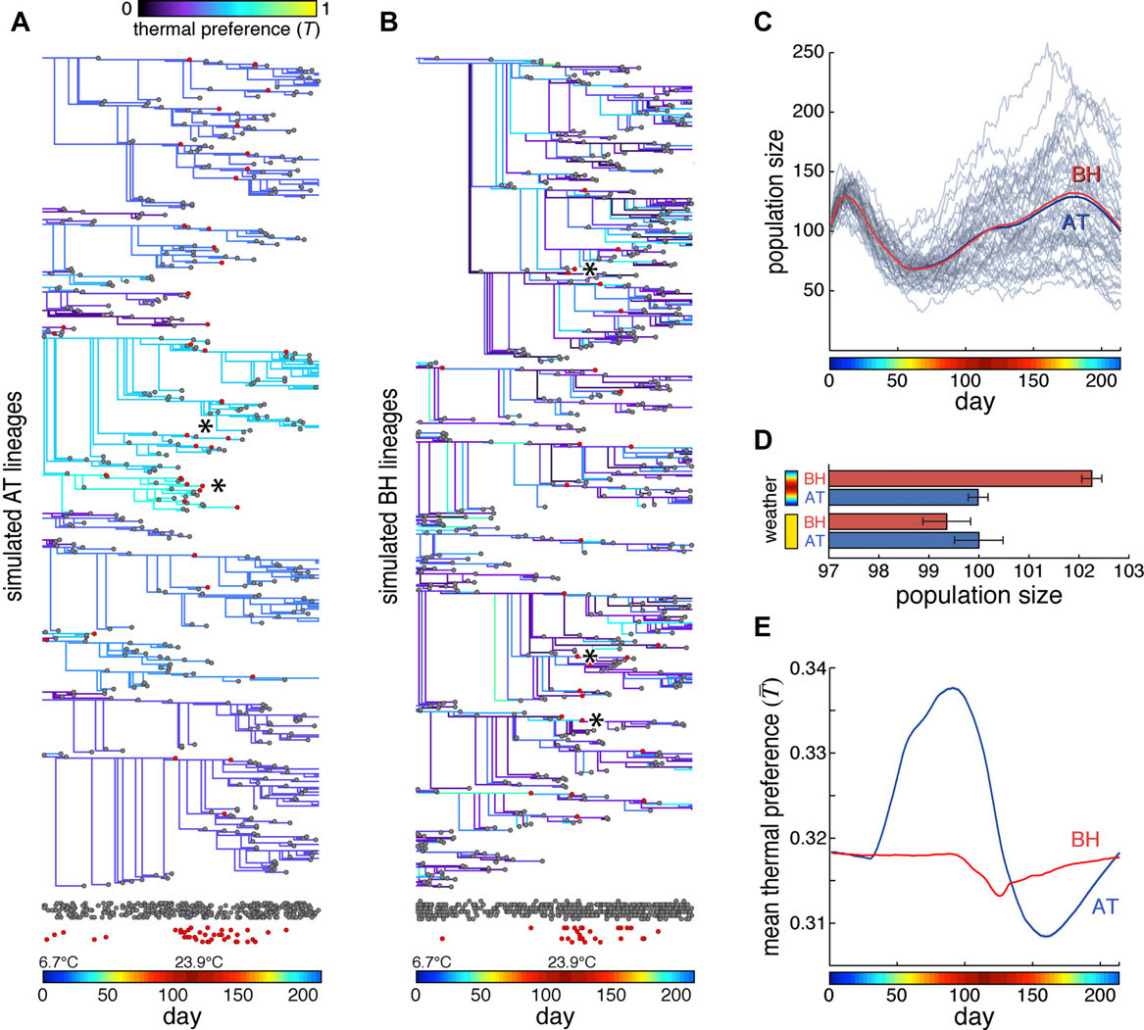
Performance of the bet‐hedging and adaptive tracking versions of the stochastic model. (A) Subset of simulated lineages from one run of the model, under the AT strategy. Branch points indicate the birth of new flies; colors indicate thermal preference index; gray dots indicate death events for reasons other than thermal experience‐dependent mortality (due to parameter δ); red dots indicate death events due to thermal experience‐dependent mortality. Rows of dots at bottom are projected from above for comparison, with random *y*‐scatter for visibility. Asterisks indicate thermal experience‐dependent death events associated with high summer temperatures in warm‐preferring lineages. Temperature at each day is indicated by the colored bar here and in all other panels. (B) As in (A), but for the BH strategy. (C) The mean performance of a bet‐hedging (BH; red line) and adaptive tracking (AT; blue) version of the model over time. Gray lines represent a sampling of 100 individual simulated seasons. (D) Mean final population size produced by each version of the model for either constant average weather (yellow) or seasonal weather (colored bar). Error bars are ±1 SE of the mean; *n* = 40,000 simulations per group. (E) Mean thermal preference index of the population over time for each version of the model. Shaded regions (barely wider than plot lines) are ±1 SE of the mean.

We simulated a stochastic (agent‐based) implementation of this model, tracking 100 individual flies experiencing the average seasonal temperature fluctuations (National Oceanic and Atmosphere Administration [NOAA] Climate Normals 2013) of a typical fly breeding season in Boston, Massachusetts, lasting approximately from April 1 to October 31 (Fig. 3). We implemented two versions of the model. (1) For the adaptive tracking strategy (AT; Fig. 3A), the thermal preference index of new flies equaled that of their mother. (2) For the bet‐hedging strategy (BH; Fig. 3B), the preference of each new fly was drawn at random from a beta distribution fitting the observed behaviors (mean thermal preference index = 0.32 and SD 0.13; Fig. 1C). The initial population of all simulations also followed this distribution, irrespective of strategy.

We measured fitness by calculating the population size at the end of the breeding season compared to the beginning. On average, the BH strategy outperformed the AT strategy by just over 2% (Fig. 3C, D, *P* < 0.0001 by *t*‐test), an effect that is completely absent (and nonsignificantly reversed, *P* = 0.64 by *t*‐test) in simulations of constant seasonal temperatures. The reason for the greater population growth of flies using BH is evident in an inspection of the average thermal preference index of the fly population across the breeding season (Fig. 3E). (The average preference changes even under BH due to temperature‐dependent shortening of the life span of warm‐seeking individuals.) In the AT strategy, the cool spring selects for warm‐preferring flies because their progeny will develop to maturity more quickly. However, at the onset of summer, the selection is reversed in favor of cool‐preferring flies, which have a longer overall life span. Once the direction of selection switches, the BH strategy begins to outperform the AT strategy, because AT responds to even transient selective pressures by shifting the population mean.

### INDIVIDUAL PHOTOTACTIC PREFERENCE IS NOT HERITABLE

**Figure 4:**
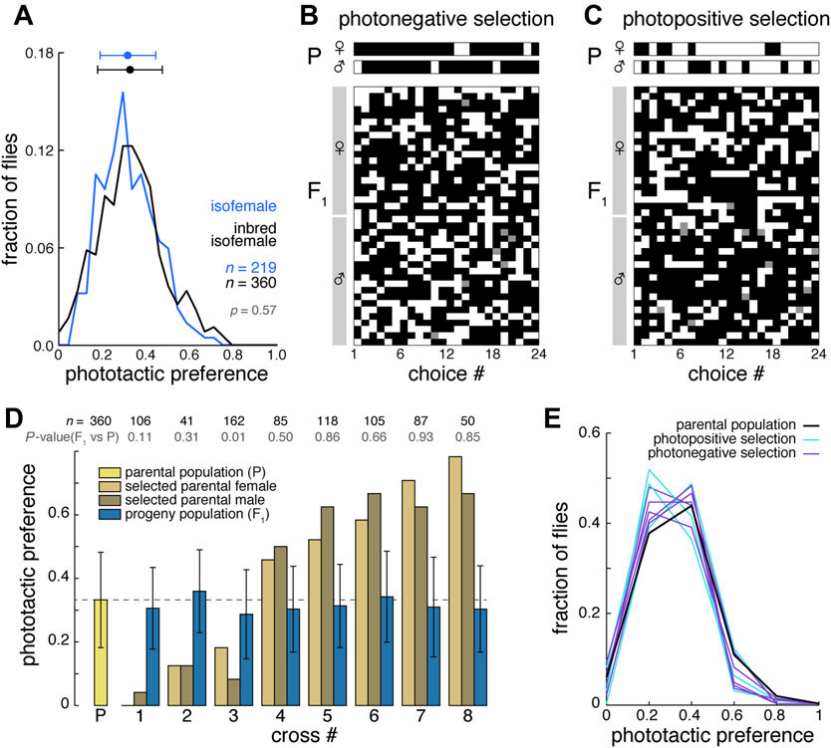
Individual phototactic preference is not heritable. (A) Observed histogram of the phototactic preference across individual CamA flies (blue) and inbred‐CamA flies (black). Points and bars represent the distribution mean and ± 1 SD. *P* values from two‐sample Kolmogorov–Smirnov test comparing each progeny distribution to the parental distribution. (B) Representative samples of the phototactic scores of a shade‐preferring male and female (top) and the phototactic scores of their resulting progeny (bottom). Each row represents an individual fly's phototactic preference over time. White boxes indicate lit choice, black boxes indicate shaded choice, and gray boxes a missing value. (C) As in (B), but for light‐preferring parents and their progeny. (D) Phototactic indices for strongly biased shade‐ or light‐preferring individuals (tan and brown bars) and their resultant progeny (dark blue bars). The dashed line and yellow bar indicate the original pool of animals from which strongly biased individual parents were selected. Numbers above bars indicate sample size, with *P* values from KS test uncorrected for multiple comparisons. Error bars are ±1 one SD. (E) Histograms of phototactic preferences within the respective progeny (D).

The model establishes that bet‐hedging is a plausible explanation for the behavioral diversity seen experimentally in thermotactic and phototactic preference. However, if the observed individuality we see truly represents bet‐hedging, then the differences in preference between individual flies are probably not due to genetic polymorphisms or transgenerational epigenetic effects, which would be heritable. This hypothesis generates two predictions: (1) reducing genetic diversity by inbreeding a polygenic stock should have no effect on the breadth of its behavioral distribution, and (2) the progeny of light‐preferring (or shade‐preferring) parents should exhibit the same distribution of behaviors as the entire parental generation, not their specific parents. (These predictions were tested in the phototactic paradigm because of its higher throughput and our use of its parameter values in the model.) We compared the behavioral distribution of our polygenic isofemale CamA line with that of the line “inbred‐CamA” that was inbred by sibling‐pair matings for 10 generations. Inbreeding had no significant effect on the mean or variance of the behavioral distribution (Fig. 4A). Using inbred‐CamA, we set up multiple crosses comprising a male and a virgin female that both either prefer the shade or the light (Fig. 4B, C). If their individual photopreferences are due to genetic polymorphisms between flies, then their progeny should have a correspondingly shifted mean photopreference relative to the original population. However, we found there was no difference in the mean photopreferences of broods derived from shade‐preferring parents versus light‐preferring (Fig. 4B–E). Using Fisher's selection estimator of heritability (*h* ^2^ = *R*/*S*), we estimated *h* ^2^ = −0.026, with a SE of 0.048. Thus, heritable polymorphisms determine at most a small component of each individual's behavior, consistent with a bet‐hedging strategy. Moreover, the distributions of brood photopreferences were indistinguishable from the parental distribution, in variance as well as mean (Fig. 4D,E).

### DETERMINISTIC MODEL SHOWS THAT THE BET‐HEDGING ADVANTAGE IS POPULATION SIZE INVARIANT

**Figure 5:**
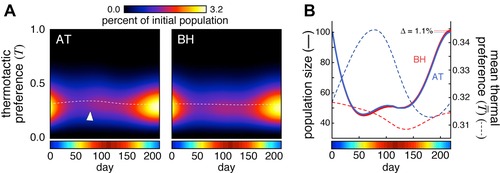
Performance of BH and AT using a difference equation implementation of the model. (A) Abundance of flies as a function of thermal preference index and time for AT and BH strategies under the difference equation model. Arrowhead indicates adaptive thermal positivity during the spring. Dashed white line indicates the mean thermotactic preference. (B) Population size (solid lines) and mean thermal preference index (dashed lines) over time of BH (red) and AT (blue) versions of the difference equation model.

The heritability intrinsic to the AT strategy means that in a finite population simulation (such as in our model population of 100 virtual flies; Fig. 3) the mean thermal preference index of the population can vary significantly from replicate to replicate due to the stochastic nature of the model (Fig. 3C). AT may lock in maladaptive thermal preference indices due to drift, and the rate at which this happens depends critically on the simulated population size (Wright 1931). Because it was arbitrary to simulate 100 animals, and effective population sizes in the wild are unknown (and perhaps far too large to simulate efficiently, Karasov et al. 2010), we developed a difference equation version of the model, in which the population size was effectively infinite and immune to stochastic effects (see Supporting Information for details). In this implementation, subpopulations of flies with specific thermal preference indices were determined by a set of difference equations (see Methods). The difference equation versions of the BH and AT strategies performed similarly to the simulations of individuals (Fig. 5A, B), with BH outperforming AT by 1.1% by the end of the summer, and the AT model undergoing two selective sweeps of opposite direction.

Using this variant of the model, we confirmed that populations utilizing either a BH or AT strategy performed best with intermediate levels of variability (Fig. S3; for these analyses, we relaxed the constraint of matching simulated variability to experimentally observed variability). Performance diminished when variability was too low or too high, supporting the hypothesis that the observed thermotactic and phototactic preference variability is adaptive. The qualitative results of this model are robust to most assumptions, but sensitive, as expected, to seasonal weather conditions and the range of temperatures accessible by behavioral choices (Table S2). The model is qualitatively sensitive to the mean thermal preference index value, which is not surprising, because altering this value means flies are mismatched to their life‐history trade‐off optimum. An AT strategy allows them to adaptively counter this mismatch.

### INCORPORATING HISTORICAL WEATHER DATA INTO THE MODEL

**Figure 6:**
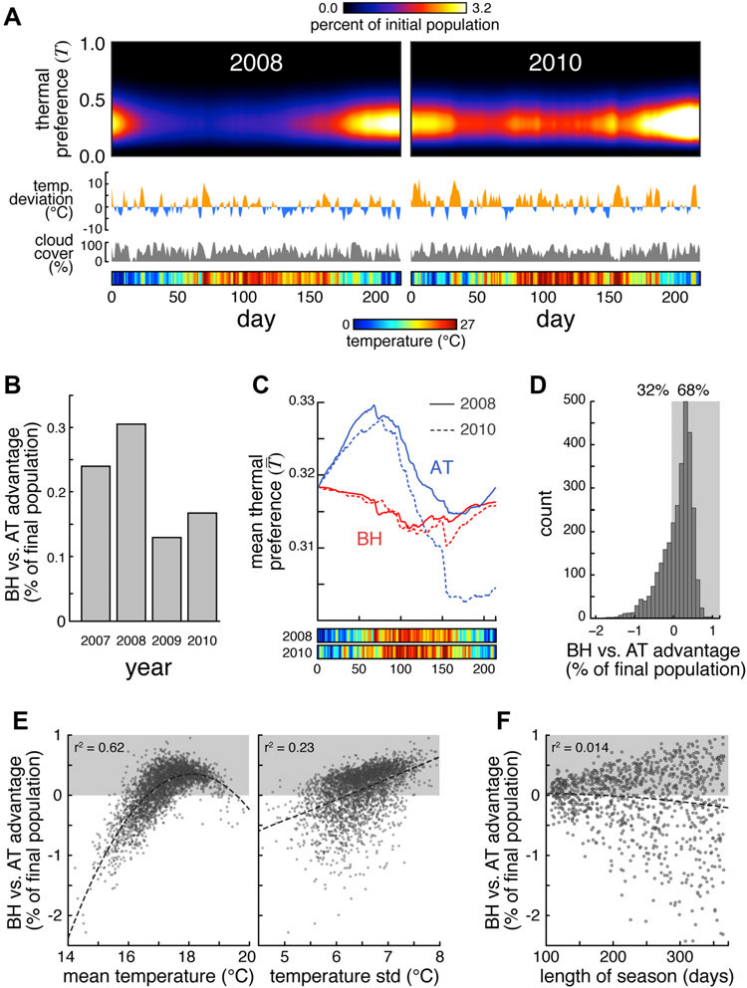
BH versus AT in historical and modeled breeding seasons. (A) Abundance of flies as a function of thermal preference index and time in the BH version of the difference equation model, applied to historical weather data (temperatures and cloud cover) from 2008 and 2010. Orange and blue traces indicate temperature deviation from daily normals. Gray traces indicate daily cloud cover percentage. Colored bar indicate the daily mean temperature. (B) BH versus AT advantage as a percent of the final population versus year ((pop_BH_ − pop_AT_)/pop_AT_ × 100). (C) Mean thermal preference indices for AT (blue) and BH (red) versions of the real weather difference equation model for 2008 (solid lines) and 2010 (dashed lines). Color bars as in (A). (D) Histogram of BH versus AT advantage as a percent of final population using the difference equation model across 3000 simulated seasons. Shaded region indicates the simulations in which BH outperformed AT. (E) Scatterplot of BH versus AT advantage versus mean temperature (left panel) or the SD of the temperature (right panel), across 3000 simulated seasons. Shaded region indicates the simulations in which BH outperformed AT. *r* ^2^ Values reflect quadratic fits (dashed lines). (F) Scatterplot of BH versus AT advantage versus breeding season length, across 1000 simulated weather seasons. Shaded region and *r* ^2^ value as in E.

To test the effects of daily temperature fluctuations and cloud cover, we ran the difference equation model against historic weather data collected in Boston, Massachusetts (NOAA Climate Normals 2013; Fig. 6A). The temperature in each day of the simulation was taken from actual historical data from that day, on a year‐by‐year basis. Cloud cover was implemented by assuming that the temperature difference available for flies to respond to (i.e., between sun and shade) each day was proportional to the average cloud cover of that day. Not surprisingly, reducing the temperature difference available to flies (due to cloud cover) reduced the magnitude of the advantage of the BH strategy (to around 0.2% for years 2007–2010; Fig. 6B). We initially thought that short‐term heat waves (or cold spells) might be enough to confer an advantage to bet‐hedging, but no clear conclusions about the impact of short‐term fluctuations could be drawn from this historical data. However, it was clear that some years were more conducive to bet‐hedging than others. For example, in 2010 the BH advantage was comparatively low (Fig. 6B). The weather that year was consistently warmer than in the others, particularly in the spring and fall (Fig. 6A, C), exerting a comparatively uniform selective pressure for cool‐seeking, thereby reducing the advantage of bet‐hedging. Consequently, the AT population exhibited a more consistent trend of decreasing mean thermal preference index across the entire year (Fig. 6C), although overall BH still outperformed AT.

### MEAN TEMPERATURE AND TEMPERATURE RANGE ARE MOST PREDICTIVE OF THE BH VERSUS AT ADVANTAGE

We developed statistical models of the daily temperature fluctuations and cloud cover that allowed us to simulate realistic random breeding seasons, and systemically tested the factors favoring the BH and AT strategies. Across 3000 random seasons, BH outperformed AT 68% of the time (Fig. 6D). We examined numerous metrics describing the simulated seasons (Fig. S4) and found two in particular that were predictive of the magnitude of the BH versus AT advantage (Fig. 6E): the temperature mean and SD. BH outperformed AT when the season has a typical temperature, while exceptionally hot or cold seasons favored the AT strategy. Additionally, AT performs poorly during “high variance” seasons (those with cold springs and falls, and hot summers) because it engenders large, lagged fluctuations in genotype frequency.

We also analyzed the effects of shorter or longer breeding seasons by compressing or stretching random temperature and cloud cover histories into seasons ranging from 107 to 365 days (Fig. 6F). The mean relative advantage of BH versus AT did not depend on season length, however, the variance of BH advantage increased with season length. Only long seasons exhibited strong advantages for either BH or AT, presumably because increasing the number of generations per season increases the potential for adaptation, whether it be productive or counterproductive. The shortest seasons exhibited little difference between BH and AT.

### GLOBAL CLIMATE CHANGE IS PREDICTED TO SHIFT EVOLUTIONARY STRATEGY FROM BH TO AT

**Figure 7:**
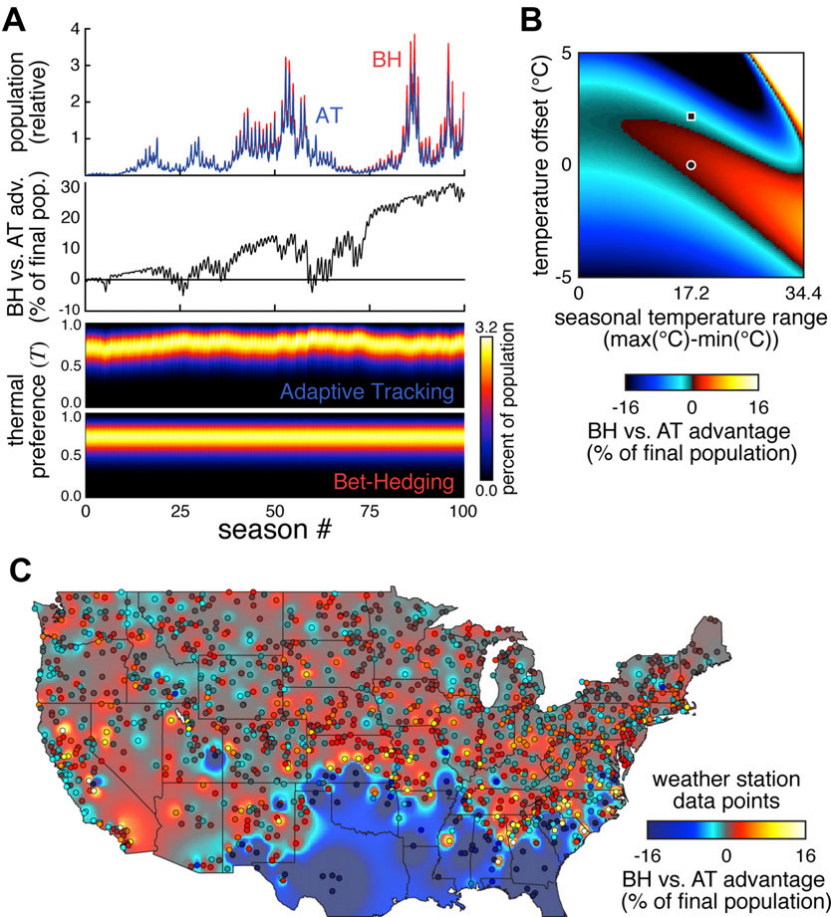
Climatic and geographic variation in BH versus AT advantage. (A) Relative population sizes for the BH (red) and AT (blue) versions of the model (top) and cumulative BH versus AT advantage (middle) over the same 100 random simulated seasons. Bottom panel shows the corresponding abundance of flies as a function of thermal preference index and time, across 100 seasons, for each strategy. (B) Phase space of BH versus AT advantage as a function of the two most predictive metrics. Color indicates magnitude of the advantage. Circle indicates current state, while the square indicates the state if the average temperature were to increase 2°C. (C) Geographic map of BH versus AT advantage. Datapoints correspond to specific NOAA weather stations; background coloration is interpolated. See Supporting Information for details.

Across the 3000 random seasons, the BH versus AT advantage never exceeds approximately 1% per season, but could drop as low as approximately −2% in some seasons (Fig. 6D). Despite the longer negative tail in this distribution, the small advantage of BH over AT in most summers quickly accumulated across simulations of multiple sequential seasons (Fig. 7A), indicating this strategy was highly favored on longer timescales. However, we found that an increase of only 2°C to the mean seasonal temperature was sufficient to change the evolutionary dynamic in favor of adaptive tracking (Fig. 7B). Conservative models of global climate change predict increases in this range in the Boston area by the end of the century (Meehl et al., 2007). Thus, while seasonal weather fluctuations generally favor bet‐hedging in thermal preference behavior, climate change will likely cause a phase shift in the evolutionarily optimal strategy toward adaptive tracking.

### GEOGRAPHICAL VARIATION IN BH VERSUS AT ADVANTAGE

Lastly, we considered to what extent the BH versus AT advantage we saw with Boston weather data was location specific. We ran the model using mean daily temperature data from more than 1400 weather stations across the continental United States (NOAA Climate Normals 2013) and compared the performance of the BH and AT strategies (Fig. 7C). Our model predicts substantial regional variation in the optimal strategy. In most locations, BH maintains a small advantage. In the deep south, where the breeding season is yearlong, allowing more time for adaptation, BH performance is much worse than AT. However, the temperature extremes and shortened breeding season of regions just north (or at higher elevation in the southern Appalachians) renders BH strongly advantageous. This is consistent with the observation that long breeding season can strongly favor either AT or BH (Fig. 6F). Consistently, the short breeding seasons of higher latitudes and the Rocky Mountains favor neither AT nor BH strongly. AT appears to be favored along the Pacific coast, which is characterized by low temperature fluctuations.

## Discussion

Here, we explored whether a bet‐hedging strategy could explain the large observed variation in temperature preference in *Drosophila*, as measured in phototactic and thermotactic paradigms. We find that in the face of fluctuating seasonal temperature selective pressures, adaptive tracking (in which progeny inherit the thermal preference index of their parent) always lags; by the time the population has adapted to the cool springtime with increased warm preference, summer arrives. By contrast, the population grows faster if the behavioral preference of individual flies is nonheritable so that there are always spring‐ and summer‐adapted animals being born. The bet‐hedging advantage is strongest under two conditions. (1) Highly variable temperatures (cool springs coupled to hot summers) magnify the selective pressure on the adaptive tracking population and thus produce larger counterproductive changes in genotype frequency as the temperature fluctuates throughout the season. This is consistent with the observations of seasonally fluctuating allele frequencies in flies (Bergland et al. 2014). (2) When mean temperatures are typical, the ability of the AT strategy to adaptively evolve is superfluous. In one example, the year 2010 was warmer on average, and its spring was particularly warm, reducing the seasonal temperature variability. Both of these factors gave the AT strategy a relative boost for being able to evolve, and thus reduced the overall BH advantage (Fig. 6B, C).

Beyond adaptive tracking and bet‐hedging, another major strategy for dealing with environmental heterogeneity is plasticity, in which organisms adaptively tune their phenotype in direct response to environmental fluctuations. The set of plasticity strategies can even include hybrid strategies such as the moment‐to‐moment regulation of the extent of bet‐hedging in response to environmental conditions. In the absence of constraints, such as metabolic cost or limits on achievable phenotypes, a plasticity strategy is tautologically optimal (DeWitt and Langerhans 2004), though such constraints surely exist. Generating an empirical estimate of the costs imposed on *Drosophila* in response to environmental fluctuations is beyond our capabilities.

Instead, we offer three lines of evidence suggesting plasticity cannot explain away the apparent adaptive advantages of bet‐hedging. First, we simulated flies that were able to use behavioral choices to achieve a preferred thermal experience, bounded by the environmental temperature range available between shade and sun. Varying strengths of this strategy were combined with bet‐hedging, and we found that over a wide range of strengths of plasticity, bet‐hedging continued to offer a relative advantage (Fig. S5). Second, we observed striking behavioral variation in populations of animals grown in essentially identical conditions (laboratory culture); to first approximation, there were no environmental fluctuations (e.g., variations in ambient temperature or luminance) to which a plasticity strategy could respond. Third, under conditions of convex fitness functions (i.e., with a single predominant mode of fit phenotypes), plasticity strategies can be at a disadvantage compared to bet‐hedging strategies even if they come with low costs (DeWitt and Langerhans 2004). The unimodal relationships between temperature and eclosion time and life span (Fig. 2B, C) yield a convex fitness function in our case, suggesting that plasticity may be outcompeted by bet‐hedging (or even adaptive tracking), even if it comes at a relatively low cost.

Our analysis focused on *D. melanogaster*, a species with a relatively short reproductive cycle capable of producing several generations within the breeding season. It is likely that species generating fewer generations per season (i.e., K‐selected species) would be less subject to the pitfalls of an adaptive tracking strategy because they would respond less to any temperature fluctuation. Although our model did not permit us to realistically change the life history of our simulated *Drosophila* in the context of real weather data, we were able to simulate changes in the length of the breeding season (Fig. 6F). Shorter seasons are comparable to a K‐selected life histories because they yield fewer generations per season. We found that, as hypothesized, shorter seasons reduce the difference between adaptive‐tracking and bet‐hedging strategies, whereas long seasons can favor either strategy depending on other factors (i.e., Fig. 6E).

This modeling highlights the importance of population‐level properties, namely the amount of variation and the heritability of that variation. Population‐level traits touch on the topic of group selection (Wilson and Wilson 2008), and indeed aspects of bet‐hedging were sometimes conflated with group selection in the literature (Hopper 1999). However, our models do not directly address this controversial issue because they have no reliance on specific population structures (the concept of which largely evaporates when considering nonheritable traits). Importantly, selection still operates, in all implementations of our model, at the level of the individual.

Two avenues for future investigation emerge from our results. First, flies captured and assayed at different time points throughout the season should show differences in their mean thermotactic preference (Fig. 3E), that reflect their mode of inheritance. Specifically, flies using an AT strategy and caught in the early summer would be comparatively warm‐seeking, whereas flies using a BH strategy would be comparatively cool‐preferring at the height of the summer, when the high temperature selectively shortens the life span of warm‐seeking individuals. However, analysis of behavior across the breeding season must consider seasonal changes in allelic frequencies (Bergland et al. 2014).

Second, flies from locales with large seasonal weather changes (e.g., Boston, MA) may have greater behavioral variation than those from milder, less varying climates (e.g., coastal central California; Fig. 7C). This prediction plays out on a variety of spatial scales, the largest being a latitudinal cline in the east and mid‐west where southern climates favor AT and northern climates BH. This prediction is consistent with recent experiments showing that northern strains of *Drosophila subobscura* are more resistant to high intensity fluctuating thermal stress, but more sensitive to prolonged (but milder) constant offset conditions (Castañeda et al. 2015). Further experiments are needed to test these hypotheses, as other groups have found no latitudinal signal across several measures of thermal tolerance and plasticity in Australian *Drosophila simulans* (van Heerwaarden, et al. 2014). Moreover, both of these studies examined isofemale lines; examination of isogenic lines would more directly permit the detection of a relationship between latitude and bet‐hedging‐derived behavioral variability.

There is also a third prediction from these models concerning the effect of climate change on these strategies. Due to incrementally increasing mean temperatures over time, AT becomes the more evolutionarily advantageous option as the organisms continually adapt to the new normal. An increase of 2°C will be sufficient to favor adaptive tracking over bet‐hedging, a change predicted to take approximately 100 years. As both phototactic (Dobzhansky and Spassky 1969) and thermotactic (Dillon et al. 2009) preferences are heritable in outbred populations, we expect that flies will be able to adapt to climate change, but not by employing bet‐hedging. Heritability of individual behaviors is a prerequisite for the evolution of AT, and it is plausible that a switch in selective pressure on strategies could increase adaptive tracking by favoring individuals with deeper developmental canalization. This would reduce the phenotypic variance associated with any single genome, and allow the distribution of genetic variation for behavioral traits to more directly determine the phenotypic distribution.

The underlying basis of individual differences in thermal preference also remains to be discovered. Many mechanisms are possible, such as variation in thermoreceptor expression or propensity to stop and rest, but our model is indifferent to the underpinnings of individual variability. The conclusions drawn from the models here are not meant to say that bet‐hedging is the sole explanation for behavioral variation. However, we have found that under the constraint of experimental data on the magnitude of behavioral variability between individuals, and with a minimal set of assumptions, bet‐hedging appears to be a more adaptive explanation of behavioral variation than deterministic genetic heterogeneity. Indeed, we believe that real *Drosophila* probably use at least three strategies—bet‐hedging, adaptive tracking, and phenotypic plasticity—to optimize its survival in an uncertain world.

## DATA ARCHIVING

The doi for our data is doi: 10.5281/zenodo.32793. Data archived at: https://zenodo.org/record/32793.

## Supporting information


**Figure S1**. Persistence of individual behavioral phenotypes.
**Figure S2**. Flowchart of the stochastic agent‐based implementation of the fly life‐history model.
**Figure S3**. End‐of‐season population size, as a function of phenotypic variance.
**Figure S4**. Relative performance of bet‐hedging versus adaptive tracking, as a function of the strength of a concurrent plasticity strategy.
**Figure S5**. BH versus AT advantage versus various seasonal measures.
**Table S1**. Weather variables simulated in each implementation of the model, and associated values of the fit birth and death rate parameters.
**Table S2**. Assessment of model qualitative robustness to various parameters and assumptions.

## References

[evo12813-bib-0001] Ashburner, M. , and J. N. Thompson . 1978. The laboratory culture of *Drosophila* Pp.100–109 *in* Ashburner and Wright, eds. The genetics and biology of *Drosophila*. Academic Press, London.

[evo12813-bib-0002] Ashburner, M. , K. G. Golic , and R. S. Hawley . 2005 *Drosophila*: a laboratory handbook (Pp. 162–164). Cold Spring Harbor Laboratory Press, Cold Spring Harbor, NY.

[evo12813-bib-0003] Ayroles, J. F. , S. M. Buchanan , C. Jenney , K. Skutt‐Kakaria , J. Grenier , A. Clark , D. Hartl , and B. L. de Bivort . 2015 Behavioral idiosyncrasy reveals genetic control of phenotypic variability. Proc. Natl. Acad. Sci. USA 112:6706–6711. 25953335 10.1073/pnas.1503830112 PMC4450409

[evo12813-bib-0004] Bergland, A. O. , E. L. Behrman , K. R. O'Brien , P. S. Schmidt , and D. A. Petrov . 2014 Genomic evidence of rapid and stable adaptive oscillations over seasonal time scales in *Drosophila* . PLoS Genet. 10:e1004775. doi:10.1371/journal.pgen.1004775. 25375361 10.1371/journal.pgen.1004775 PMC4222749

[evo12813-bib-0005] Buchanan, S. M. , J. S. Kain , and B. L. de Bivort . 2015 Neuronal control of locomotor handedness in *Drosophila* . Proc. Natl. Acad. Sci. USA 112:6700–6705. 25953337 10.1073/pnas.1500804112 PMC4450378

[evo12813-bib-0006] Castañeda, L. E. , E. L. Rezende , and M. Santos . 2015 Heat tolerance in *Drosophila subobscura* along a latitudinal gradient: contrasting patterns between plastic and genetic responses. Evolution. doi:10.1111/evo.12757. 69:2721–2734 26292981 10.1111/evo.12757

[evo12813-bib-0007] Cohen, D. 1966 Optimizing reproduction in a randomly varying environment. J. Theor. Biol. 12:119–129. 6015423 10.1016/0022-5193(66)90188-3

[evo12813-bib-0008] Condon, C. , A. Acharya , G. J. Adrian , A. M. Hurliman , D. Malekooti , P. Nguyen , M. H. Zelic , and M. J. Angilletta . 2015 Indirect selection of thermal tolerance during experimental evolution of *Drosophila melanogaster* . Ecol. Evol. 5:1873–1880. 26140203 10.1002/ece3.1472 PMC4485968

[evo12813-bib-0009] DeWitt, T. J. , and B. R. Langerhans . 2004 Integrated solutions to environmental heterogeneity: theory of multimoment reaction norms. Oxford Univ. Press, New York.

[evo12813-bib-0010] DeWitt, T. J. , A. Sih , and D. Sloan Wilson . 1998 Costs and limits of phenotypic plasticity. Trends Ecol. Evol 13:77–81. 21238209 10.1016/s0169-5347(97)01274-3

[evo12813-bib-0011] Digby, P. S. Factors affecting the temperature excess of insects in sunshine . 1955 Factors affecting the temperature excess of insects in sunshine. J. Exp. Biol. 32:279–298.

[evo12813-bib-0012] Dillon, M. E. , G. Wang , P. A. Garrity , and R. B. Huey . 2009 Review: thermal preference in *Drosophila* . J. Therm. Biol. 34:109–119. 20161211 10.1016/j.jtherbio.2008.11.007 PMC2714919

[evo12813-bib-0013] Dobzhansky, T. , and B. Spassky . 1969 Artificial and natural selection for two behavioral traits in *Drosophila pseudoobscura* . Proc. Natl. Acad. Sci. USA 62:75–80. 5253666 10.1073/pnas.62.1.75 PMC285957

[evo12813-bib-0014] Dochtermann, N. A. , T. Schwab , and A. Sih . 2015 The contribution of additive genetic variation to personality variation: heritability of personality. Proc. Biol. Sci. 282:20142201. 25392476 10.1098/rspb.2014.2201 PMC4262176

[evo12813-bib-0015] Haccou, P. , and Y. Iwasa . 2002 Optimal mixed strategies in stochastic environments. Theor. Pop. Biol. 47:212–243.

[evo12813-bib-0016] Hopper, K. R. Risk‐spreading and bet‐hedging in insect population biology. 1999 Annu. Rev. Entomol. 44:535–560. 15012381 10.1146/annurev.ento.44.1.535

[evo12813-bib-0017] Huang, Y. , S. Wright , and A. Agarwal . 2014 Genome‐wide patterns of genetic variation within and among alternative selective regimes. PLOS Genet. 10(8):e1004527. 25101783 10.1371/journal.pgen.1004527 PMC4125100

[evo12813-bib-0018] Izquierdo, J. I. 1991 How does *Drosophila melanogaster* overwinter? Entomol. Exp. Appl. 59:51–58.

[evo12813-bib-0019] Kain, J. S. , C. Stokes , and B. L. de Bivort . 2012 Phototactic personality in fruit flies and its suppression by serotonin and white. Proc. Natl. Acad. Sci. USA 109:19834–19839. 23150588 10.1073/pnas.1211988109 PMC3511718

[evo12813-bib-0020] Karasov, T. , P. W. Messer , and D. A. Petrov . 2010 Evidence that adaptation in *Drosophila* is not limited by mutation at single sites. PLoS Genet. 6:e1000924. 20585551 10.1371/journal.pgen.1000924 PMC2887467

[evo12813-bib-0021] Kawecki, T. J. 2000 The evolution of genetic canalization under fluctuating selection. Evolution 54:1–12. 10937177 10.1111/j.0014-3820.2000.tb00001.x

[evo12813-bib-0022] Levy, S. F. , N. Ziv , and M. L. Siegal . 2012 Bet hedging in yeast by heterogeneous, age‐correlated expression of a stress protectant. PLoS Biol. 10:e1001325. 22589700 10.1371/journal.pbio.1001325 PMC3348152

[evo12813-bib-0023] Lewontin, R. 1959 On the anomalous response of *Drosophila pseudoobscura* to light. Am. Nat. 93:321–328.

[evo12813-bib-0024] Martin, T. L. , and R. B. Huey . 2008 Why “suboptimal” is optimal: Jensen's inequality and ectotherm thermal preferences. Am. Nat. 171:E102–E111. 18271721 10.1086/527502

[evo12813-bib-0025] Meehl, G. A. , T. F. Stocker , W. D. Collins , P. Friedlingstien , and A. T. Gaye . Global climate projections In Climate change 2007: the physical science basis. Contribution of working group I to the fourth assessment report of the intergovernmental panel on climate change. Pp. 749–844 *in* Solomon, S., D. Qin, M. Manning, Z. Chen, M. Marquis, K.B. Averyt, M. Tignor and H.L. Miller (eds.) Cambridge Univ. Press, Cambridge, U.K.

[evo12813-bib-0026] Miquel, J. , P. R. Lundgren , K. G. Bensch , and H. Atlan . 1976 Effects of temperature on the life span, vitality and fine structure of *Drosophila melanogaster* . Mech. Ageing Dev. 5:347–370. 823384 10.1016/0047-6374(76)90034-8

[evo12813-bib-0027] Müller, J. , B. A. Hense , T. M. Fuchs , M. Utz , and C. Pötzsche . 2013 Bet‐hedging in stochastically switching environments. J. Theor. Biol. 7:144–157. 10.1016/j.jtbi.2013.07.017 23899941

[evo12813-bib-0028] Murren, C. , J. Auld , H. Callahan , C. Ghalambor , C. Handelsman , M. Heskel , J. Kingsolver , H. Maclean , J. Masel , H. Maughan , et al. 2015 Constraints on the evolution of phenotypic plasticity: limits and costs of phenotype and plasticity. Heredity. doi:10.1038/hdy.2015.8. 115:293–301 25690179 10.1038/hdy.2015.8 PMC4815460

[evo12813-bib-0029] National Oceanic and Atmosphere Administration (NOAA) 1981–2010 Climate Normals. 2013 Available at http://www.ncdc.noaa.gov/data‐access/land‐based‐station‐data/land‐based‐datasets/climate‐normals/1981‐2010‐normals‐data. Accessed September 6, 2014.

[evo12813-bib-0030] Parry, D. A. 1951 Factors determining the temperature of terrestrial arthropods in sunlight. J. Exp. Biol. 28:445–462.

[evo12813-bib-0031] Pittendrigh, C. S. Adaptation, natural selection and behavior 1958 Pp. 390–416 *in* Behavior and evolution. Yale Univ. Press, New Haven, CT.

[evo12813-bib-0032] Rockwell, R. F. , and M. B. Seiger . 1973 Phototaxis in *Drosophila*: a critical evaluation. Am. Sci. 61:339–345.

[evo12813-bib-0033] Ryu, W. S. , and A. D. Samuel . 2002 Thermotaxis in *Caenorhabditis elegans* analyzed by measuring responses to defined thermal stimuli. J. Neurosci. 22:5727–5733. 12097525 10.1523/JNEUROSCI.22-13-05727.2002 PMC6758190

[evo12813-bib-0034] Sasaki, A. , and S. Ellner . 1995 The evolutionary stable phenotype distribution in a random environment. Evolution 492:337–350. 10.1111/j.1558-5646.1995.tb02246.x 28565011

[evo12813-bib-0035] Scott, J. P. 1943 Effects of single genes on the behavior of *Drosophila* . Am. Nat. 77:184–190.

[evo12813-bib-0036] Seiger, M. B. , L. A. Seiger , and J. A. Kertesz . 1983 Photoresponse in relation to experimental design in sibling sympatric species of *Drosophila* . Am. Midl. Nat. 109:163–168.

[evo12813-bib-0037] Simons, A. M. 2011 Modes of response to environmental change and the elusive empirical evidence for bet hedging. Proc. Biol. Sci. 278:1601–1609. 21411456 10.1098/rspb.2011.0176 PMC3081777

[evo12813-bib-0038] Sultan, S. E. 2000 Phenotypic plasticity for plant development, function and life history. Trends Plant Sci. 5:537–542. 11120476 10.1016/s1360-1385(00)01797-0

[evo12813-bib-0039] Svardal, H. , C. Rueffler , and J. Hermisson . 2011 Comparing environmental and genetic variance as adaptive response to fluctuating selection. Evolution 65:2492–2513. 21884052 10.1111/j.1558-5646.2011.01318.x

[evo12813-bib-0040] Tobler, R. , J. Hermisson , and C. Schlötterer . 2015 Parallel trait adaptation across opposing thermal environments in experimental *Drosophila melanogaster* populations. Evolution. doi:10.1111/evo.12705. 69:1745–1759 26080903 10.1111/evo.12705 PMC4755034

[evo12813-bib-0041] van Heerwaarden, B. , R. F. H. Lee , J. Overgaard , and C. M. Sgrò . 2014 No patterns in thermal plasticity along a latitudinal gradient in *Drosophila simulans* from eastern Australia. J. Evol Biol. 2:2541–2553. 10.1111/jeb.12510 25262984

[evo12813-bib-0042] Waddington, C. H. , B. Woolf , and M. M. Perry . 1954 Environment selection by *Drosophila* mutants. Evolution 8:89–96.

[evo12813-bib-0043] Wilson, D. S. , and E. O. Wilson . 2008 Evolution For the good of the group. Am. Sci. 96:380–389.

[evo12813-bib-0044] Wright, S. 1931 Evolution in Mendelian populations. Genetics 16:97–159. 17246615 10.1093/genetics/16.2.97 PMC1201091

