# Supplementary material for: Variability in thermal and phototactic preferences in Drosophila may reflect an adaptive bet‐hedging strategy
Source: Evolution. 2015 Nov 30;69(12):3171–85. doi: 10.1111/evo.12813 (PMC5063146; doi:10.1111/evo.12813)
Supplement: Supplementary file 1 — Figure S1. Persistence of individual behavioral phenotypes. Figure S2. Flowchart of the stochastic agent‐based implementation of the fly life‐history model. Figure S3. End‐of‐season population size, as a function of phenotypic variance. Figure S4. Relative performance of bet‐hedging versus adaptive tracking, as a function of the strength of a concurrent plasticity strategy. Figure S5. BH versus AT advantage versus various seasonal measures. Table S1. Weather variables simulated in each implementation of the model, and associated values of the fit birth and death rate parameters. Table S2. Assessment of model qualitative robustness to various parameters and assumptions. [file EVO-69-3171-s001.pdf]

Online Supporting Information for:

# Bet-hedging, seasons and the evolution of behavioral diversity in *Drosophila*


Jamey S. Kain[1], Sarah Zhang[1], Jamilla Akhund-Zade[2,3], Aravinthan D. T. Samuel[2,4], Mason Klein[2,4,5], Benjamin L. de Bivort[1,2,3,*]

[1]Rowland Institute at Harvard, Cambridge, Massachusetts 02142, USA

[2]Center for Brain Science, Harvard University, Cambridge, Massachusetts 02138, USA

[3]Department of Organismic and Evolutionary Biology, Harvard University, Cambridge, Massachusetts 02138, USA

[4]Department of Physics, Harvard University, Cambridge, Massachusetts 02138, USA

[5]Department of Physics, University of Miami, Coral Gables, Florida 33124, USA

* Author for correspondence

Author email addresses: resolute@gmail.com, zhang.sar@gmail.com, jakhundzade@g.harvard.edu, samuel@physics.harvard.edu, klein@physics.miami.edu, debivort@oeb.harvard.edu

**Supporting Methods**

## Difference equation model

The difference equation model was:

$$N_j(t) = N_j(t-1) * (1 - \delta - \Delta(j)) + \sum_i (N_i(t-1) * F(i) * P_j(i) * \beta)$$

$$\Delta(j) = 1/(0.04074\tau(j)^2 - 28.356\tau(j) + 506.2)$$

$$\tau(j) = \text{Temp}(t) + j * 5°C * \text{Sky}(t)$$

$$F(i) = 1 - D(i)/L(i)$$

$$L(i) = 1/(\Delta(i) + \delta)$$

$$D(i) = 0.2306\tau_D(i)^2 - 11.828\tau_D(i) + 158.34$$

$$\tau_D(i) = \frac{1}{t_{D_2} - t_{D_1}} \sum_{t'=t_{D_1}}^{t_{D_2}} (\text{Temp}(t') + i * 5°C * \text{Sky}(t'))$$

$$t_{D_1} = \lfloor 0.5 + \max(1, t - L(i)/2) \rfloor$$

$$t_{D_2} = \lfloor 0.5 + \min(t, t_{D_1} + D(\tau'_D)) \rfloor$$

$$\tau'_D = \frac{1}{t'_{D_2} - t_{D_1}} \sum_{t''=t_{D_1}}^{t'_{D_2}} \text{Temp}(t'')$$

$$t'_{D_2} = \min(t, t_{D_1} + 21)$$

$$P_j(i) = \begin{cases} \text{BetaPDF}(i, a, b) : h^2 = 0 \\ \begin{cases} 1 : i = j \\ 0 : i \neq j \end{cases} \quad : h^2 = 1 \end{cases}$$

Here, $N_j(t)$ is the number of flies alive at time $t$ with thermal preference index $j$. $\Delta(j)$ is the rate at which flies die due to thermal experience-dependent mortality as a function of $j$. $\tau(j)$ is the effective temperature experienced by flies with thermal preference index $j$, with Temp($t$) indicating temperature and Sky($t$) indicating respectively the temperature and cloud cover fraction at time $t$. The summation term in $N_j(t)$ indicates the number of flies born at time $t$ with thermal preference index $j$, born from parents with thermal preference index $i$, which depends on the population sizes of flies with thermal preference index $i$ at time $t$-1($N_i(t-1)$), the fraction of each of those parental subpopulations which are fertile ($F(i)$) and the probability densities of parental thermal preference index ($P_j(i)$) conditioned on the thermal preference index of the progeny ($j$), and given the alternative BH vs AT strategies. ($P_j(i)$ is coded as a matrix with probability entries in row $j$, column $i$. For strategy AT, it is the identity matrix; for strategy BH, every row of $P_j(i)$ equals the beta-fit distribution from Figure 1C.) $F(i)$ depends on the ratio of development time $D(i)$ to total lifespan $L(i)$ of flies with thermal preference index $i$. $D(i)$ depends on the effective temperature experienced by parents (as this determines egg laying site) during development $\tau_D(i)$ which we approximate as the mean effective temperature across a range starting at time $t$ minus half the typical lifespan, and ending $D(\tau'_D)$ days later (bounded by the time endpoints of the simulation). Development time is dependent on integrated temperature, which in turn depends on the length of development, given temperature's temporal fluctuation. So the calculation of $D(\tau'_D)$ reflects one level of recursion in the calculation of this feedback. $\tau'_D$ is calculated as the average temperature from $t'_{D1}$ through 21 days later, an interval approximating half a typical lifespan. The results of the difference equation model are very

robust to the choice of the intervals in this recursion approximation, as well as the number of recursive levels implemented.

In comparisons of populations with differing initial thermal preference index distributions, we could not use the same values of δ and *β* for both conditions. Thus, an approach of fitting those parameters to satisfy the constraint of constant population size from beginning to end of the breeding season would not work - both populations would tautologically have identical populations at the end of the season. In these cases, we calibrated δ and *β* using a different assumption: identical mean population sizes across the breeding season, thus allowing the final population size to vary, and allowing us to assess relative growth rates. The other fitting assumption, that the mean thermal preference index did not evolve, was invoked in all cases.

In simulations of sequential seasons, the mean thermal preference index of the initial population of each season was set to the mean of population at the end of the previous season, but the variance was reset to match the empirical data. In geographic simulations, breeding seasons were defined as all days between the first day of the year in which temperatures reach 6.5°C and the first day when mean temperatures fall below 10°C, the same thresholds used in the Boston season. The non-parity in these values reflect our understanding that the first thaw suffices to end diapause while the first frost is sufficient to trigger it. The specific predictions associated with some stations are sensitive to these bounds, but the overall geographic patterns are not. The $\beta$ and $\delta$ parameters were fit independently for each station automatically using a hill-climbing algorithm. Included stations were chosen at random from the 7500 stations in the NOAA data set, however, the algorithm was unable to fit the model parameters for some stations in very hot regions, i.e. some of the deep south and the Mojave desert, so station geographic sampling is not unbiased. Background interpolation in geographic maps was done pixel by pixel using the function $b_{x,y} = \sum_i b_i * w_i / \sum_i w_i$, i.e. the average of all stations indexed by *i* and weighted by $w_i$, where $w_i = ((x - x_i)^2 + (y - y_i)^2)^{-2/3}$, i.e. inverse Euclidean distance from the pixel (*x*,*y*) to station *i* raised to the third power. This exponent was chosen to ensure a sharp drop-off with distance from the stations, but is otherwise arbitrary.

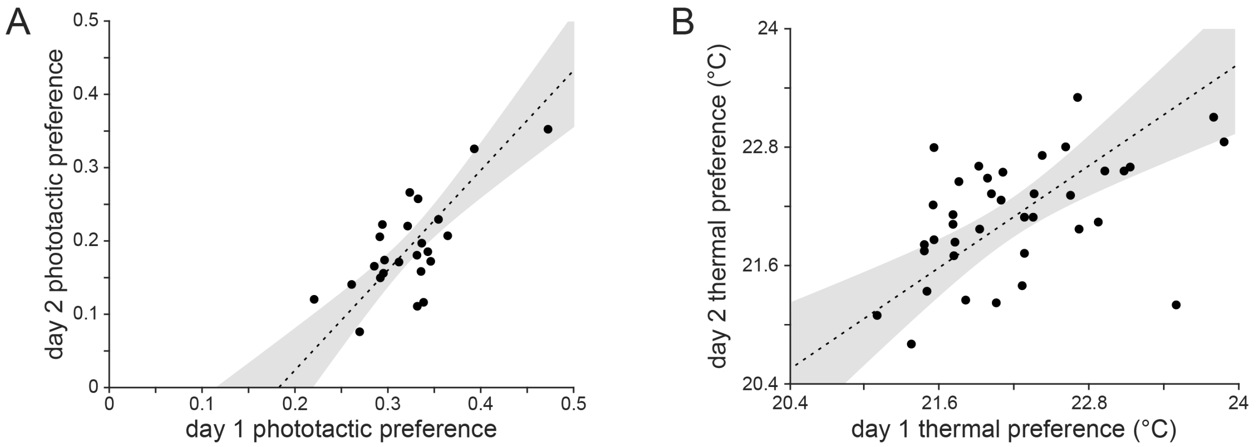


**Figure S1** – Persistence of individual behavioral phenotypes. A) Scatter plot of phototactic preference on day 2 versus phototactic preference on day 1. Flies were assayed for 40 hours continuously. Their position with respect to a phototactic gradient was recorded every 10 minutes. Daily preference values were calculated as the average of all observations in the first 24 hours (day 1) and all the points in the last 16 hours of the recording (day 2). Points are individual flies; dotted line is the best geometric mean regression fit; shaded region is the 95% confidence interval on the fit as determined by bootstrap resampling. Pearson $r$ = 0.71, $n$ = 24, $p < 0.0001$. B) As in (A) for thermal preference. Rather than continuous recording, in the thermal persistence experiment, thermal preference was measured as in all other thermal experiments, over four hours, and fly identity was maintained across successive days by individual housing. Regression fits as in A. Pearson $r$ = 0.48, $n$ = 37, $p$ = 0.002. Genotypes of the inbred flies tested here are $w^{1118}$ and DGRP line #796 respectively.

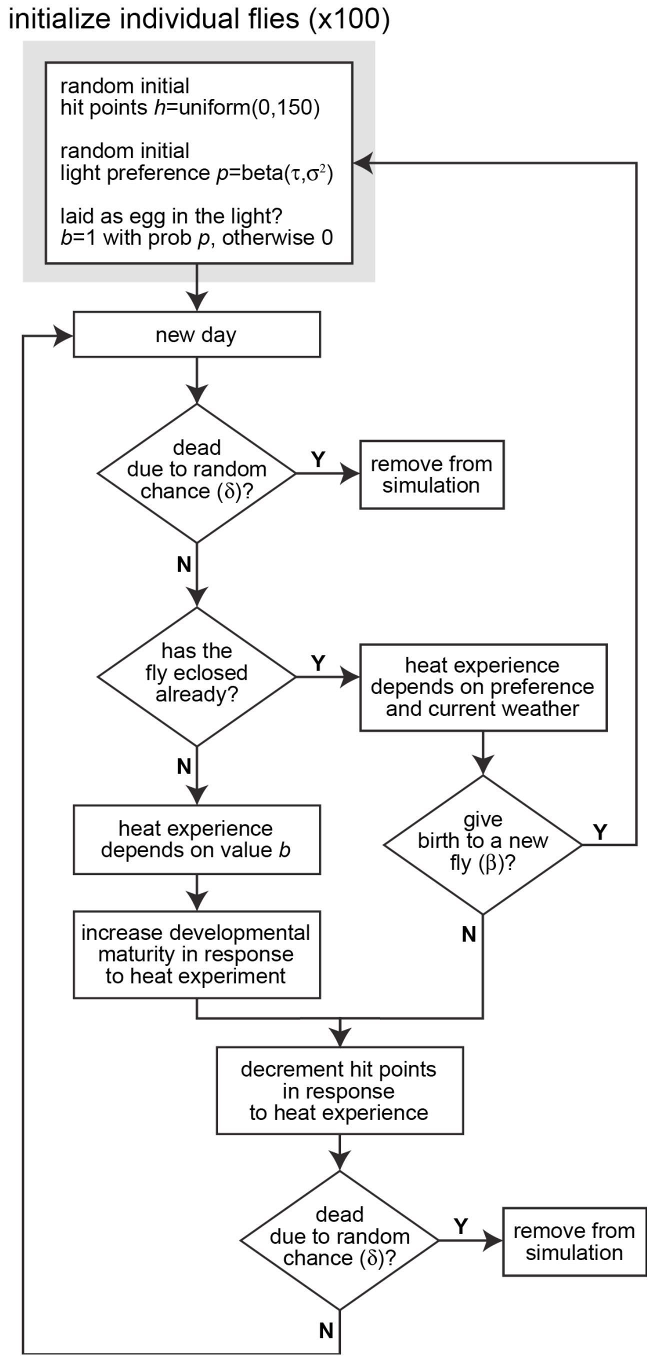


**Figure S2** – Flowchart of the stochastic agent-based implementation of the fly life history model. See Results for additional explanation.

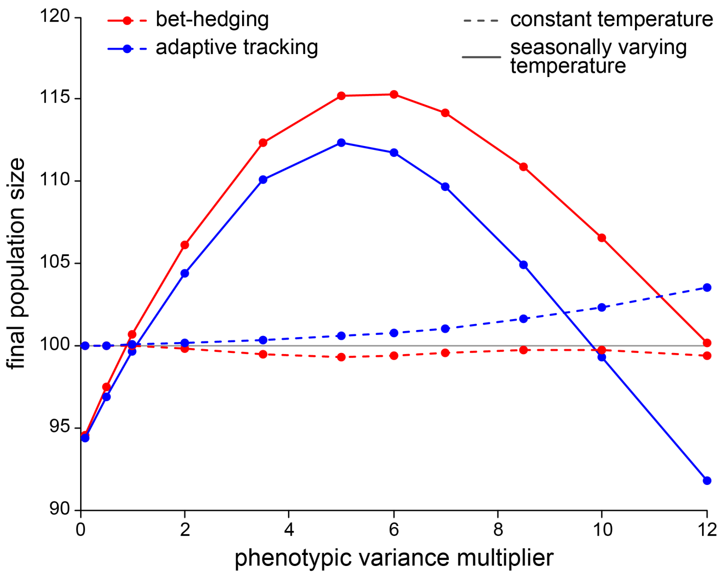


**Figure S3** – End-of-season population size, as a function of phenotypic variance. Solid lines reflect performance under seasonally varying temperatures (following the mean daily deviations for Boston, MA). Dotted lines reflect performance in constant temperature seasons. Red indicates bet-hedging populations, blue adaptive tracking. In all conditions the initial phenotypic distribution followed a beta distribution with mean equal to the experimental mean thermal preference, and variance equal to the experimental variance multiplied by the multiplier indicated on the x-axis.

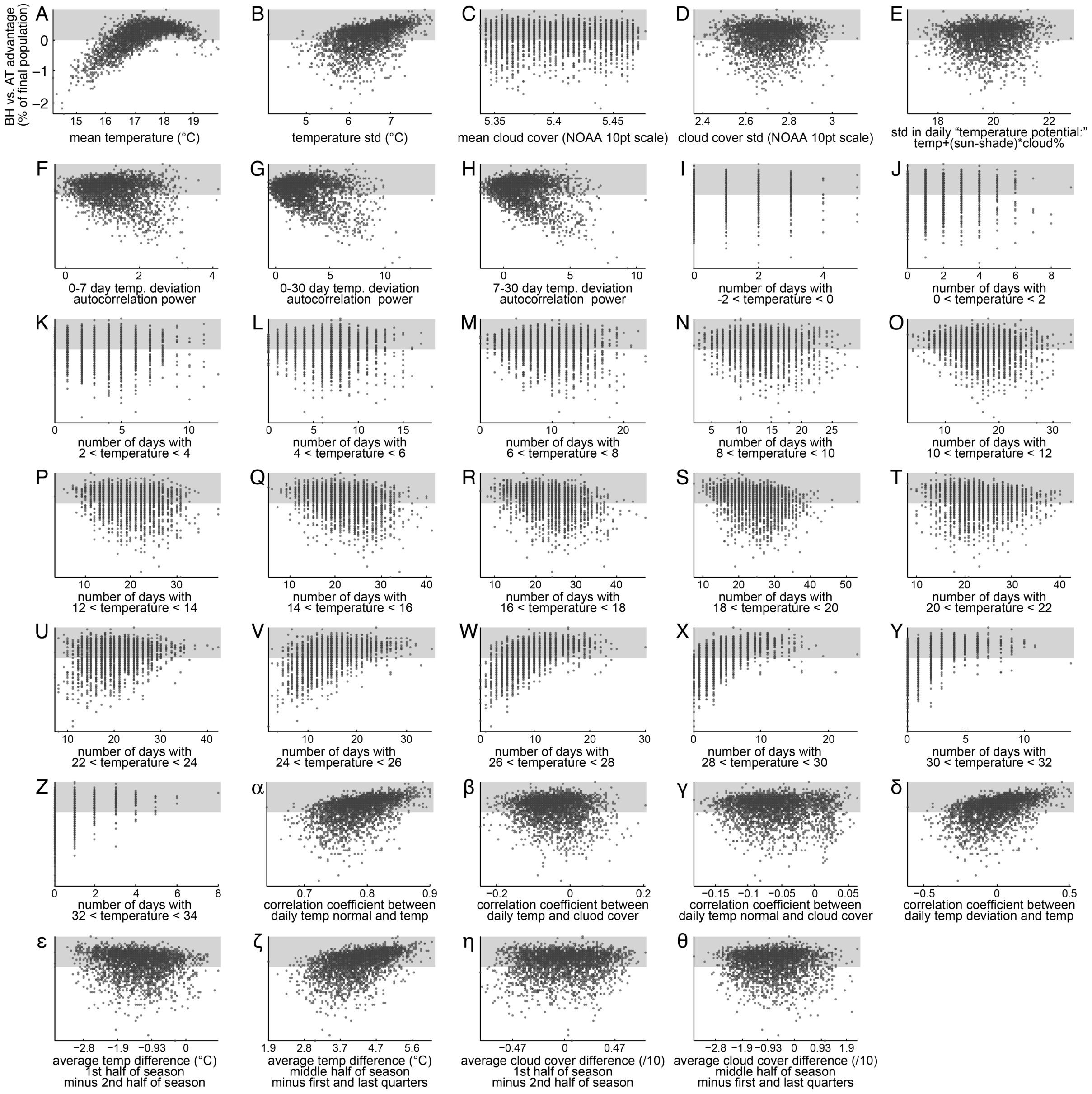


**Figure S4** – BH versus AT advantage versus various seasonal measures. All Y-axes as in first panel. F-H were calculated by summing the normalized autocorrelation vector of the daily temperature deviations for the specified range of offsets. Most measures showing clear relationships with BH-AT advantage reduce to either mean temperature (A) or temperature standard deviation (B). As examples: Seasons with many days of moderate temperature (R, S) correspond to seasons of low temperature standard deviation. Conversely, seasons with more very high temperature days (X-Z) correspond to hot years (A). Seasons with greater correlation between daily temperature and temperature normal ($\alpha$, $\gamma$) have more extreme temperature ranges, corresponding to (B).

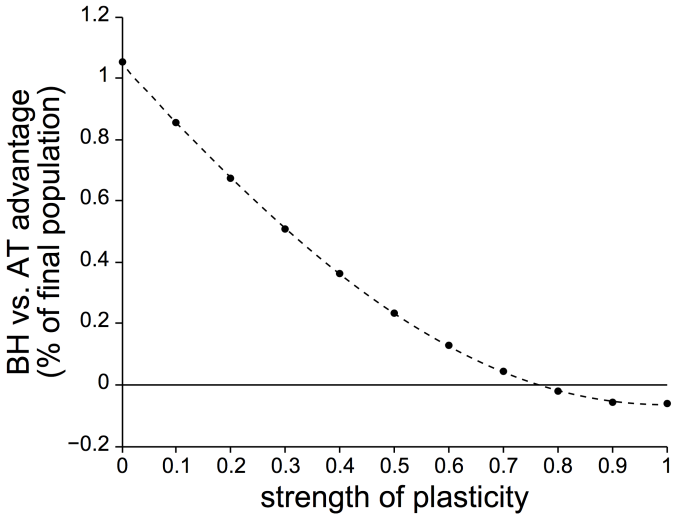


$$ATD = \begin{cases} -shadeDiff & \text{if } S - T < -shadeDiff \\ S - (T + x * shadeDiff * cloudCover) & -shadeDiff < S - T < shadeDiff \\ shadeDiff & S - T > shadeDiff \end{cases}$$

**Figure S5** – Relative performance of bet-hedging vs. adaptive tracking, as a function of the strength of a concurrent plasticity strategy. Top) Simulated flies experienced temperatures corresponding to the weighted average of their baseline strategy (either BH or AT) and a plasticity strategy in which they could choose to rest either in the shade, or the sun, in whatever portion brought their thermal experience closest to the species-wide preferred temperature (25°C). X-axis corresponds to the relative weighting of the plasticity strategy, with 0 indicating entirely BH or AT, and 1 indicating entirely the plastic strategy. Bottom) We simulated this strategy by assuming that the thermal experience of flies on a given day was *T* + *x* * *shadeDiff* * *cloudCover* + *p* * *ATD*, where *T* is the daily in-shade temperature, *x* is the animal's thermal preference, *shadeDiff* is the shade vs. sunlight temperature difference (7°C), cloud cover is the fraction of cloud cover (0-10 in NOAA coding, divided by 10 in our calculations), *p* is the strength of plasticity, and *ATD* is the "achievable temperature delta" (the temperature offset attainable by behavioral choices, equation given above).

**Table S1** – Weather variables simulated in each implementation of the model, and associated values of the fit birth and death rate parameters.

| Figure Panel(s) | Model Type | Variables Simulated | $\beta$ | $\delta$ |
|---|---|---|---|---|
| 2A-E | stochastic | daily temperature normals | 0.1062 | 0.0435 |
| 2D | stochastic | constant seasonal temperature | 0.0366 | 0.0203 |
| 4A-B | difference equation | daily temperature normals | 0.04480243 | 0.012755 |
| 5A-C | difference equation | daily temperature normals<br>historical daily temperature deviations<br>historical daily cloud cover fractions | 0.05388375 | 0.013635 |
| 5D-E | difference equation | daily temperature normals<br>simulated daily temperature deviations<br>simulated daily cloud cover fractions | 0.05388375 | 0.013635 |
| 6A | difference equation | daily temperature normals<br>simulated daily temperature deviations<br>simulated daily cloud cover fractions<br>100 sequential seasons | 0.04661 | 0.01168 |
| 6B | difference equation | daily temperature normals | 0.04480243 | 0.012755 |
| 6C | difference equation | daily temperature normals from 1469 different locations | varies | varies |

**Table S2** – Assessment of model qualitative robustness to various parameters and assumptions. * indicates robustness in the relative performance of bet-hedging and adaptive tracking, but unrealistic output otherwise, such as rapidly expanding populations when $\beta$ is increased on its own. Bold figures indicate conditions with BH out performing AT at the end of the season. Numbers in parentheses indicate default values. All parameters or assumptions were changed singly, while holding all others at their default values.

| Parameter or Assumption | Robust or Sensitive | Evidence |
|---|---|---|
| thermal preference mean (0.3183) | sensitive | TPM = 0.16 … BH vs AT = -47%<br>TPM = 0.26 … BH vs AT = -11%<br>TPM = 0.38 … BH vs AT = -1.7% |
| thermal preference variance (0.0162) | robust | TPV = 0.012 … BH vs AT = **0.82%**<br>TPV = 0.032 … BH vs AT = **1.7%** |
| birth rate parameter ($\beta$) (0.04480243) | robust* | $\beta$ = 0.040 … BH vs AT = **1.2%**<br>$\beta$ = 0.060 … BH vs AT = **0.05%** |
| death rate parameter ($\delta$) (0.012755) | robust* | $\delta$ = 0.008 … BH vs AT = **0.62%**<br>$\delta$ = 0.016 … BH vs AT = **0.047%** |
| shade temperature difference (7°C) | robust | STD = 4°C … BH vs AT = **0.27%**<br>STD = 10°C … BH vs AT = **1.5%** |
| time to eclosion vs. temperature = $M(T)$ | robust | $M(T) = 0.23T^2 – 11.8T + 168$ (+10d offset)<br>… BH vs AT = **1.5%**<br>$M(T) = 90 – 3T$ (linear, decreasing)<br>… BH vs AT = **0.24%**<br>$M(T) = 100000T^{-3}$ (asymptotically decreasing)<br>… BH vs AT = **1.5%** |
| adult lifespan vs. temperature = $A(T)$ | robust | $A(T) = 0.41T^2 – 28.4T + 606$ (+100d offset)<br>… BH vs AT = **0.035%**<br>$A(T) = 130 – 3T$ (linear, decreasing)<br>… BH vs AT = **0.17%**<br>$A(T) = 120 – 0.12T^2$ (concave-down, decreasing)<br>… BH vs AT = **0.22%** |
| immature fly thermal experience determined by parental preference | robust | Stochastic simulation run with immature fly thermal experience determined by their own preference. Results statistically indistinguishable. Not applicable to difference equation model. |
| flies overwinter as adults | robust | Stochastic simulation run with initial fly ages either uniformly distributed > maturity age, or 0. Results statistically indistinguishable. Not applicable to difference equation model. |
| seasonal weather conditions | varies | Figures 2D, 5, 6, S5 |
| length of breeding season | robust | Figure 5F |
| inclusion of plastic behavioral responses | robust | Figure S4 |
